# Development and validation of a nomogram for predicting depressive symptoms in dentistry patients: A cross-sectional study

**DOI:** 10.1097/MD.0000000000037635

**Published:** 2024-04-05

**Authors:** Jimin Zhang, Zewen Huang, Wei Wang, Lejun Zhang, Heli Lu

**Affiliations:** a Department of Stomatology, No. 903 Hospital of PLA Joint Logistic Support Force (Xi Hu Affiliated Hospital of Hangzhou Medical College), Hangzhou, China; b Department of Special Education and Counselling, The Education University of Hong Kong, Tai Po, China; c Department of Psychology, The Education University of Hong Kong, Tai Po, China; d School of Psychology, South China Normal University, Guangzhou, China; e Department of Psychosomatic Medicine, The Second Affiliated Hospital of Nanchang University, Nanchang, China.

**Keywords:** cross-sectional study, dentistry patients, depressive symptoms, nomogram

## Abstract

Depressive symptoms are frequently occur among dentistry patients, many of whom struggle with dental anxiety and poor oral conditions. Identifying the factors that influence these symptoms can enable dentists to recognize and address mental health concerns more effectively. This study aimed to investigate the factors associated with depressive symptoms in dentistry patients and develop a clinical tool, a nomogram, to assist dentists in predicting these symptoms. Methods: After exclusion of ineligible participants, a total of 1355 patients from the dentistry department were included. The patients were randomly assigned to training and validation sets at a 2:1 ratio. The LASSO regression method was initially employed to select highly influrtial features. This was followed by the application of a multi-factor logistic regression to determine independent factors and construct a nomogram. And it was evaluated by 4 methods and 2 indicators. The nomograms were formulated based on questionnaire data collected from dentistry patients. Nomogram^2^ incorporated factors such as medical burden, personality traits (extraversion, conscientiousness, and emotional stability), life purpose, and life satisfaction. In the training set, Nomogram^2^ exhibited a Concordance index (C-index) of 0.805 and an Area Under the Receiver Operating Characteristic Curve (AUC) of 0.805 (95% CI: 0.775–0.835). In the validation set, Nomogram^2^ demonstrated an Area Under the Receiver Operating Characteristic Curve (AUC) of 0.810 (0.768–0.851) and a Concordance index (C-index) of 0.810. Similarly, Nomogram^1^ achieved an Area Under the Receiver Operating Characteristic Curve (AUC) of 0.816 (0.788–0.845) and a Concordance index (C-index) of 0.816 in the training set, and an Area Under the Receiver Operating Characteristic Curve (AUC) of 0.824 (95% CI: 0.784–0.864) and a Concordance index (C-index) of 0.824 in the validation set. Net Reclassification Improvement (NRI) and Integrated Discrimination Improvement (IDI) indicated that Nomogram^1^, which included oral-related factors (oral health and dental anxiety), outperformed Nomogram^2^. We developed a nomogram to predict depressive symptoms in dentistry patients. Importantly, this nomogram can serve as a valuable psychometric tool for dentists, facilitating the assessment of their patients’ mental health and enabling more tailored treatment plans.

## 1. Introduction

Oral diseases have a significant detrimental impact on human health, affecting a large number of people worldwide.^[1]^ Furthermore, poor oral health can potentially lead to irreversible consequences such as oral cancer. From a societal perspective, oral diseases impose a considerable economic burden on countries or regions and strain healthcare systems.^[2]^ At an individual level, dentistry patients often endure chronic pain, significantly diminishing their quality of life.^[3]^ It is important to note the complex interplay between oral health and mental health.^[4,5]^ On one hand, many dentistry patients experience frequent anxiety during dental visits.^[6]^ In some cases, this anxiety can evolve into a long-lasting mental illness, profoundly impacting patients’ lives.^[7]^ On the other hand, patients with dental diseases may subjectively amplify their pain sensations, irrespective of the extent of their oral lesions at the time.^[8]^ For example, studies have found that individuals with burning mouth syndrome are more prone to depression or anxiety.^[9]^ Therefore, it is crucial to pay attention to the mental health status of patients with oral diseases.

Depression is a complex, multifactorial condition influenced by various factors such as gender, personality, values, and socioeconomic status.^[10]^ It poses a significant threat to both physical and mental health, with severely depressed patients being at risk of suicide.^[11]^ Even mild depression has been associated with multiple negative outcomes, as individuals diagnosed with mild depression are highly susceptible to diminished quality of life.^[12]^ Depression often manifests as physical pain and discomfort, with studies indicating that depressed patients may experience headaches, backaches, and stomachaches.^[13]^ Moreover, depression negatively affects sleep patterns, energy levels, and daily activities.^[14–16]^ Notably, oral-related factors, such as dental anxiety and oral health, have a strong relationship with depression. Firstly, depression is closely associated with dental disorders,^[17]^ and patients with oral diseases or poor oral health (e.g., missing teeth, tooth loss, and dental caries) are at an increased risk of developing depression.^[18]^ Additionally, common oral health outcomes have been linked to various mental illnesses, including anxiety disorders, schizophrenia, and bipolar disorder.^[19]^ Secondly, dental anxiety is considered a risk factor that impacts the mental health of patients with oral diseases.^[20]^ Individuals with dental anxiety often experience high levels of stress associated with dental treatment.^[21,22]^ Furthermore, deteriorating dental conditions can lead to feelings of shame and low self-esteem, further exacerbating dental anxiety.^[20]^ These effects can have long-term negative consequences on patients’ lives and ultimately reduce their overall quality of life.^[23]^ Patients with dental anxiety often face multiple challenges, including psychosomatic illnesses, unemployment, and difficulties in interpersonal relationships, all of which contribute to the development of depressive symptoms.^[24]^

In China, dental services are very expensive and unaffordable for low-income families.^[25]^ As a result, dental visits are not an routine service and the vast majority of people only choose to visit a dentist when they are faced with an obvious oral problem.^[26]^ We believe that patients who visit the dentistry department usually suffer from relatively serious oral diseases and are representative of the group with dental problems. Therefore, patients who visit the dentistry department often have significant oral diseases and can be considered representative of the population with dental problems. Consequently, dentistry patients may be at a higher risk of developing depressive symptoms. Despite the increased risk of depression in dentistry patients due to their oral diseases and dental anxiety, limited research has systematically investigated the factors influencing depression in this specific population. While dentists acknowledge that some patients with oral diseases may also suffer from psychological disorders, their professional knowledge and other factors limit their ability to accurately diagnose psychological disorders in their patients.^[27]^ Hence, there is an urgent need for a simple and reliable model to help dentists and patients estimate the risk of depressive symptoms.

Drawing upon the previously developed nomograms for predicting depressive symptoms in other populations, this study aimed to analyze data from patients visiting the dentistry clinic to develop an effective predictive model for depressive symptoms specifically in dentistry patients and validate its performance. Additionally, this study emphasized several aspects that should be considered in incorporating the nomogram prediction model. Firstly, it is important to include as many demographic variables as possible as predictors while selecting a concise scale or questionnaire to enhance the model’s usefulness. Secondly, incorporating factors that reflect patients’ personal characteristics (e.g., personality, purpose in life, well-being, and life satisfaction) and behavioral characteristics (smoking, alcohol consumption, and physical activity) can improve the predictive performance of the model. Lastly, oral-related factors, such as dental anxiety and oral health, should be taken into account to explore their specific effects on depression in dentistry patients.

## 2. Methods

### 2.1. Patients

Between September 10 and September 30, 2022, a total of 1439 patients attending the Department of Stomatology in the 903rd Hospital of PLA were recruited for this study. To ensure an appropriate sample size, the mean sample size from previous studies on nomograms of depressive symptoms was used as a reference indicator.^[28–30]^ Therefore, a sample size larger than 404 participants was deemed suitable. The patients who agreed to participate in the study were asked to anonymously complete a web-based questionnaire by scanning a QR code. Prior to answering the questionnaire, patients were required to read and understand the consent form, which outlined the study’s purpose, potential risks and benefits, and the participants’ rights. Only those who checked the box indicating their agreement to participate were able to proceed with the questionnaire. For participants under the age of 18, both the participant and their guardian were asked to confirm their willingness to participate. The questionnaire ensured that no personally identifiable information was collected or available to the authors. To ensure complete responses, mandatory response settings were implemented, and participants could only submit the questionnaire after answering all items. Therefore, there were no missing values in the collected data. To prevent careless or invalid responses, 3 attention check items were included in the online questionnaire.^[31]^ Participants who correctly answered at least 2 attention check items were considered qualified. After excluding participants who did not meet the criteria, the final sample consisted of 1355 patients, resulting in a questionnaire validity rate of 94.16%. The age of the patients ranged from 11 to 88 years, with a mean age of 30.17 (SD: 10.35). Among the patients, 525 (38.7%) were male and 830 (61.3%) were female. The participants were randomly assigned to either the training set (n = 904) or the validation set (n = 541) in a 2:1 ratio. This study was approved by the Biomedical Research Ethics Committee of the 903rd Hospital of PLA (Research and Clinical Review No. 20220816/10/01/001).

### 2.2. Measurement tools

#### 2.2.1. Personality traits.

The Chinese version of the Ten Item Personality Inventory (TIPI-C) consists of 10 items, of which 1, 3, 5, 7, and 9 are positive scoring items and 2, 4, 6, 8, and 10 are negative scoring items.^[32]^ The entire scale contains a total of 10 questions, which measure subjects’ scores on five dimensions, which are Extroversion, Agreeableness, Conscientiousness, Emotional Stability, Openness to Experiences. The scale is based on a 7-point scale. The Cronbach’s α for this evaluation was 0.74.

#### 2.2.2. Purpose in life.

The Purpose in Life Test-Short Form (PIL-SF) consists of 4 items, which are translated into Chinese using the backflip method.^[33]^ The scale uses a 7-point scale with a total score between 4 and 28, the higher the score, the stronger the sense of purpose and meaning in life. Since the PIL-SF uses a 7-point scale, the meaning of each level is clear. Where the number 4 represents neutral, meaning neither side of the feeling, this unclear attitude represents the individual’s feeling of meaningfulness in life is also unclear. The quadratic scale can be used to classify and compare college students with different levels of meaningfulness. <4 is classified as individuals with low sense of life meaning, lacking life goals, 4 to 4.99 is classified as individuals with moderate sense of life meaning, with unclear life goals, 5 to 5.99 is classified as individuals with high sense of life meaning, with clearer life goals, and 6 and above is classified as individuals with very high sense of life meaning, with very clear life purposes in life. The Cronbach’s α for this evaluation was 0.94.

#### 2.2.3. Life satisfaction.

The Satisfaction with Life Scale^[34]^ consists of 5 questions, each of which is scored on a 7-point scale. The higher the total score, the more satisfied the individual is with his or her life. In this study, we used the Cronbach’s α to represent the reliability of the scale, and the reliability of Satisfaction with Life Scale was 0.94.

#### 2.2.4. Subjective well-being.

The Subjective Well-being Scale^[35]^consists of an item in which participants answer the question “Overall, how do you feel you are happy” and score it on a 7-point scale, with higher scores associated with higher well-being.

#### 2.2.5. Physical activity.

The Physical Activity Rating Scale (PARS-3) was used in this survey, mainly reflecting the physical activity of the subjects since the last month.^[36]^ The PARS-3 assesses the physical activity of individuals in terms of time, frequency and intensity. In the present study, we tested the reliability of this scale and the results showed that the Cronbach’s α was 0.78.

#### 2.2.6. Alcohol and tobacco use.

The Tobacco and Alcohol Use Scale is a scale that measures the frequency and quantity of smoking and alcohol consumption by subjects.^[37]^ This scale uses a 6-point scale and has 4 questions. The reliability (Cronbach’s α) of this scale was 0.70.

#### 2.2.7. Oral health.

The Oral Health Impact Profile-14 (OHIP-14) scale was developed by Slade (1997)^[38]^ on the basis of the complete version of the OHIP-49.^[39]^ The OHIP-14 scale was developed by Slade in 1997 based on the complete version of the OHIP-49, and t consists of 14 items in 4 dimensions (handicap, functional limitation, pain and comfort, disability). They have been translated into multiple languages and used in different cultural contexts, and their psychometric properties have been confirmed to be stable and reliable. Based on this, the OHIP-14 was chosen as the scale for this study. The Cronbach’s α for this evaluation was 0.95.

#### 2.2.8. Dental anxiety.

The Stouthard’s Dental Anxiety Inventory (DAI) was developed by Stouthard et al and is considered one of the more comprehensive scales for assessing dental anxiety, with a full version of 36 questions.^[40]^ Each question consists of 5 multiple-choice responses, ranging from “Disagree completely on a scale of 1” to “Agree completely on a scale of 5” to indicate the level of anxiety. The Short-form Stouthard’s Dental Anxiety Inventory (S-DAI), which retained the 3 major directions of the full version and showed that the S-DAI could also assess dental anxiety in a comprehensive manner.^[41]^ The Cronbach’s α for this evaluation was 0.94.

#### 2.2.9. Depressive symptoms.

The Patient Health Questionnaire (PHQ-9) is a brief, self-assessment tool that is often used in primary care settings for the diagnosis of mental disorders.^[42]^ It is different from other diagnostic tools in that it is based on the DSM-IV diagnostic criteria and includes 5 major components: depression, anxiety, substance abuse, eating disorders, and somatization disorders. The PHQ-9 is a scale for depression, which has been translated into several languages and is widely used as a screening tool in primary care units. It has 9 items, i.e., 9 depressive symptoms, which are: loss of cheerfulness, depressed mood, sleep disturbance, lack of energy, eating disorder, low self-esteem, difficulty concentrating, slow movement, and negative perception. The scores for each entry in the first part are as follows: 0 = not at all, 1 = a few days, 2 = more than a week, 3 = almost every day. In this study, a PHQ-9 score greater than 4 was used as a cutoff point for determining depressive symptoms, and all patients with a score greater than 4 were considered to have some degree of depressive symptoms. The Cronbach’s α for this evaluation was 0.92.

### 2.3. Statistical analysis

The online questionnaires were distributed through QR code on the WENJUANXIN platform, which provides a service akin to Amazon Mechanical Turk. Descriptive statistics for all variables and subsequent Chi-square tests, independent samples *t* tests were performed using SPSS 28.0. R-Studio was employed for the elimination of unqualified questionnaires, random grouping of the training and validation sets, the execution of LASSO regression analysis and Multivariate logistic regression analysis, and the construction and validation of Nomogram prediction models.

## 3. Results

### 3.1. Clinical characteristics

Table 1 presents the patient characteristics in the training and validation sets. There were no significant differences observed between the 2 sets regarding the probability of depressive symptoms. The occurrence of depressive symptoms was 35.7% in the training set and 33% in the validation set. Similarly, we did not discover any significant disparities in the characteristics related to depressive symptoms, such as age, gender, census, only child status, number of siblings, first dental visit, dental visit frequency, education, marital status, number of offspring, identity, medical insurance, types of medical insurance, medical burden, chronic disease, income, socioeconomic status, personality traits, life purpose, life satisfaction, subjective well-being, alcohol and tobacco usage, physical activity, oral health, and dental anxiety (Table 1).

**Table 1:** Baseline value of the training set and the validation set.

Variable	Category	Training set (n = 904)	Validation set (n = 451)	*P*
Depression symptoms	No	581 (64.3%)	302 (67%)	.358
	Yes	323 (35.7%)	149 (33%)	NA
Age		30.24 ± 10.58	30.04 ± 9.88	.911
Gender	Female	567 (62.7%)	263 (58.3%)	.131
	Male	337 (37.3%)	188 (41.7%)	NA
Census	Rural household registration	385 (42.6%)	200 (44.3%)	.577
	Urban household registration	519 (57.4%)	251 (55.7%)	NA
Only child or not	No	609 (67.4%)	319 (70.7%)	.232
	Yes	295 (32.6%)	132 (29.3%)	NA
The number of siblings	No sibling	295 (32.6%)	132 (29.3%)	.738
	One sibling	345 (38.2%)	191 (42.4%)	NA
	Two siblings	170 (18.8%)	80 (17.7%)	NA
	Three siblings	63 (7%)	33 (7.3%)	NA
	Four siblings	13 (1.4%)	7 (1.6%)	NA
	More than 4 siblings	18 (2%)	8 (1.8%)	NA
First visit to dentistry	No	425 (47%)	211 (46.8%)	.983
	Yes	479 (53%)	240 (53.2%)	NA
The number of visits to dentistry		1.51 ± 3.36	1.37 ± 3.13	.760
Education level	No-schooling	3 (0.3%)	1 (0.2%)	.756
	Elementary school	6 (0.7%)	3 (0.7%)	NA
	Junior high school	30 (3.3%)	9 (2%)	NA
	High school	66 (7.3%)	35 (7.8%)	NA
	Secondary school	45 (5%)	26 (5.8%)	NA
	Junior college	214 (23.7%)	107 (23.7%)	NA
	University	455 (50.3%)	219 (48.6%)	NA
	Master	81 (9%)	46 (10.2%)	NA
	Doctor	4 (0.4%)	5 (1.1%)	NA
Marital status	Unmarried	569 (62.9%)	278 (61.6%)	.101
	Married	329 (36.4%)	164 (36.4%)	NA
	Divorced	4 (0.4%)	8 (1.8%)	NA
	Widowed	2 (0.2%)	1 (0.2%)	NA
The number of children	No child	569 (62.9%)	278 (61.6%)	.923
	One child	56 (6.2%)	27 (6%)	NA
	Two children	179 (19.8%)	98 (21.7%)	NA
	Three children	96 (10.6%)	47 (10.4%)	NA
	Four children or more than 4 children	4 (0.4%)	1 (0.2%)	NA
Identity	The masses	666 (73.7%)	319 (70.7%)	.213
	Retired cadres working in government or public institutions	13 (1.4%)	5 (1.1%)	NA
	Military personnel	142 (15.7%)	83 (18.4%)	NA
	Family members of military personnel	24 (2.7%)	6 (1.3%)	NA
	Workers of government agencies or institutions	59 (6.5%)	38 (8.4%)	NA
Types of medical insurance	Self-financed	212 (23.5%)	106 (23.5%)	.289
	Medical insurance basic social health insurance	605 (66.9%)	292 (64.7%)	NA
	Medical insurance commercial insurance	7 (0.8%)	1 (0.2%)	NA
	Medical insurance other	80 (8.8%)	52 (11.5%)	NA
Medical burden	No burden at all	422 (46.7%)	205 (45.5%)	.908
	Basically no burden	322 (35.6%)	164 (36.4%)	NA
	A little burden	148 (16.4%)	74 (16.4%)	NA
	Very heavy burden	12 (1.3%)	8 (1.8%)	NA
Chronic disease	No	863 (95.5%)	436 (96.7%)	.363
	Yes	41 (4.5%)	15 (3.3%)	NA
Income	Less than 500 CNY	93 (10.3%)	45 (10%)	.835
	500–1000 CNY	42 (4.6%)	24 (5.3%)	NA
	1001–2000 CNY	55 (6.1%)	28 (6.2%)	NA
	2001–3000 CNY	29 (3.2%)	14 (3.1%)	NA
	3001–5000 CNY	133 (14.7%)	59 (13.1%)	NA
	5001–8000 CNY	226 (25%)	106 (23.5%)	NA
	8001–10,000 CNY	131 (14.5%)	60 (13.3%)	NA
	More than 10,000 CNY	195 (21.6%)	115 (25.5%)	NA
Socioeconomic status		5.32 ± 1.72	5.41 ± 1.77	.222
Extraversion		4.42 ± 1.2	4.39 ± 1.23	.755
Agreeableness		4.78 ± 0.99	4.77 ± 0.96	.914
Conscientiousness		4.64 ± 1.14	4.66 ± 1.15	.957
Emotional stability		4.64 ± 1.19	4.6 ± 1.21	.796
Openness to experiences		4.36 ± 1.05	4.41 ± 1.06	.379
Purpose in life		20.46 ± 5.01	20.61 ± 5.26	.655
Life satisfaction		22.88 ± 7.27	22.9 ± 7.6	.834
General well-being		5.09 ± 1.34	5.09 ± 1.37	.895
Alcohol and tobacco use		1.48 ± 0.83	1.47 ± 0.87	.492
Physical activity		17.74 ± 23.05	18.73 ± 21.93	.214
Oral health		12.91 ± 11.6	12.78 ± 11.2	.999
Dental anxiety		20.48 ± 9.29	20.24 ± 9.36	.644

### 3.2. Feature selection and construction of nomogram^1^ and nomogram^2^

**Figure 1.**
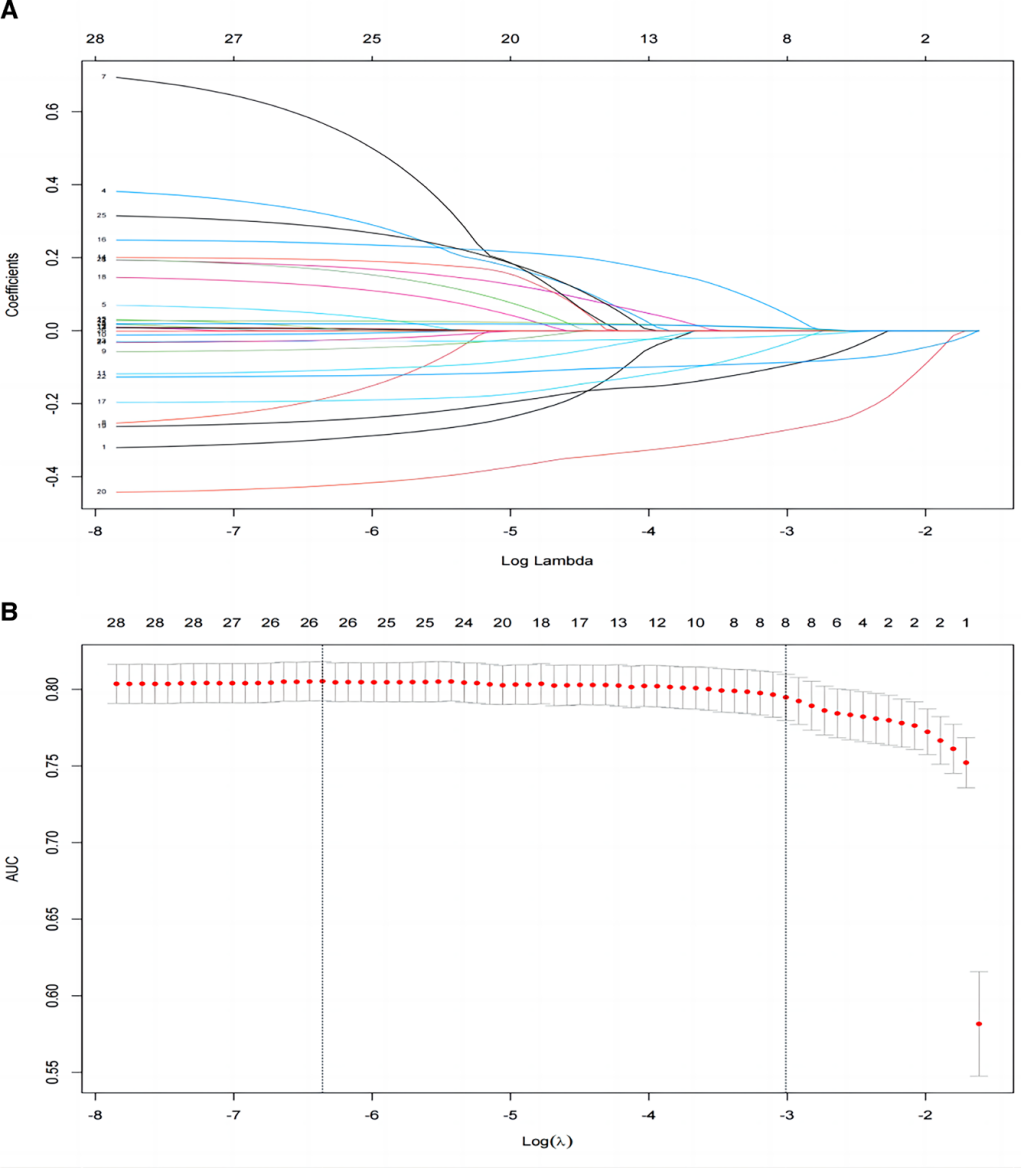
Texture feature selection using the least absolute shrinkage and selection operator (LASSO) regression model. (A) Identification of the optimal penalization coefficient lambda (λ) in the LASSO model with 10-fold cross-validation in the Training set. The plot shows the relationship between the lambda values and the corresponding cross-validated error rate, helping to select the optimal lambda for the LASSO model. (B) LASSO coefficient profiles of 8 features in the training set. The plot illustrates the trajectory of each depression symptom-related feature’s coefficient in the LASSO coefficient profiles as the lambda value changes in the LASSO algorithm. This profile helps to visualize the effect of different lambda values on feature selection and the magnitude of the coefficients associated with each feature. The figure demonstrates the use of the LASSO regression model for texture feature selection in predicting depression symptoms. It provides insights into the optimal lambda selection and the impact of lambda on the coefficients of the depression symptom-related features.

**Figure 2.**
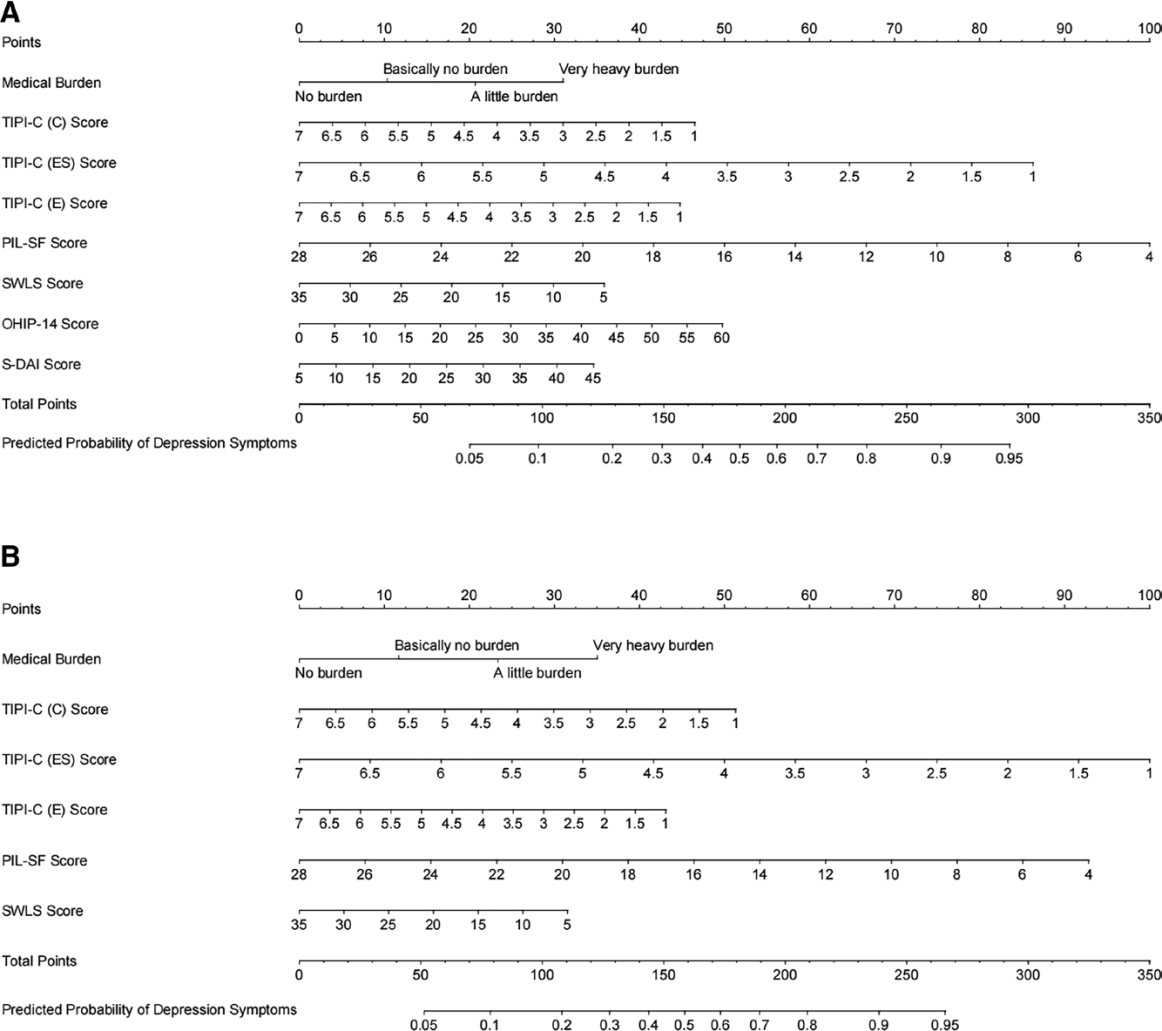
Nomogram for the prediction of depression symptoms. (A) Nomogram^1^ was constructed based on the data of the Training set. The Nomogram^1^ provides a visual representation of the predictive model for depression symptoms, incorporating various independent variables from the Training set. (B) Nomogram^2^ was constructed based on the Training set. To investigate the impact of Oral-related factors on depression symptoms, Nomogram^2^ includes 6 independent variables excluding Oral Health and Dental Anxiety. This Nomogram allows for a comparison of the predictive power of the included factors with and without the Oral-related variables. The Nomograms (Nomogram^1^ and Nomogram^2^) serve as graphical tools to estimate the probability of depression symptoms based on the included independent variables. They provide a user-friendly approach for clinicians and researchers to make predictions and understand the relative contributions of different factors in relation to depression symptoms. DAI = The Stouthard’s Dental Anxiety Inventory, OHIP-14 = The Oral Health Impact Profile-14, PIL-SF = The Purpose in Life Test-Short Form, SWLS = The Satisfaction With Life Scale, TIPI-C (A) = Ten Item Personality Inventory (Agreeableness), TIPI-C (C) = Ten Item Personality Inventory (Conscientiousness), TIPI-C (E) = Ten Item Personality Inventory (Extroversion), TIPI-C (ES) = Ten Item Personality Inventory (Emotional Stability), TIPI-C (OE) = Ten Item Personality Inventory (Openness to Experiences).

We selected 8 potential predictors from the original set of 28 texture features, based on data from 1355 patients in the primary cohort (3.5:1 ratio) using the LASSO regression model (Fig. 1A). These selected features had nonzero coefficients in the LASSO regression model. The multivariate logistic regression analysis confirmed that all 8 variables (medical burden, personality traits (extraversion, conscientiousness, and emotional stability), life purpose, and life satisfaction and oral-related factors (oral health and dental anxiety)) were significant predictors of depressive symptoms (Table 2; Fig. 1B). Consequently, these 8 independent predictors were used to construct nomogram^1^ in the training set (Fig. 2A). We also constructed a separate nomogram (nomogram^2^) excluding oral health and dental anxiety by utilizing the remaining 6 independent factors to assess the specific impact of oral-related factors on depressive symptoms (Fig. 2B).

**Table 2:** Nomogram for depression symptoms prediction.

Intercept and variable	Model 1 (Nomogram^1^)				Model 2 (Nomogram^2^)		
	OR	95% CI	*P*	OR		95% CI	*P*
Intercept	118.15	34.60–426.90	<.001	349.28		115.76–1123.58	<.001
Medical burden	1.32	1.07–1.62	.010	1.38		1.12–1.69	.002
Extraversion	0.82	0.67–0.98	.015	0.79		0.70–0.96	.014
Conscientiousness	0.81	0.70–0.96	.034	0.63		0.65–0.95	.015
Emotional stability	0.68	0.57–0.82	<.001	0.82		0.53–0.76	<.001
Purpose in life	0.90	0.86–0.93	<.001	0.90		0.86–0.94	<.001
Life satisfaction	0.97	0.94–1.00	.023	0.97		0.95–1.00	.034
Oral health	1.02	1.01–1.04	.003	NA		NA	NA
Dental anxiety	1.02	1.00–1.04	.017	NA		NA	NA
C-index							
Training set	0.816 (0.788–0.845)				0.805 (0.775–0.835)		
Validation set	0.824 (0.784–0.864)				0.810 (0.768–0.851)		

### 3.3. Assessment of nomogram^1^ and nomogram^2^ in the training set

**Figure 3.**
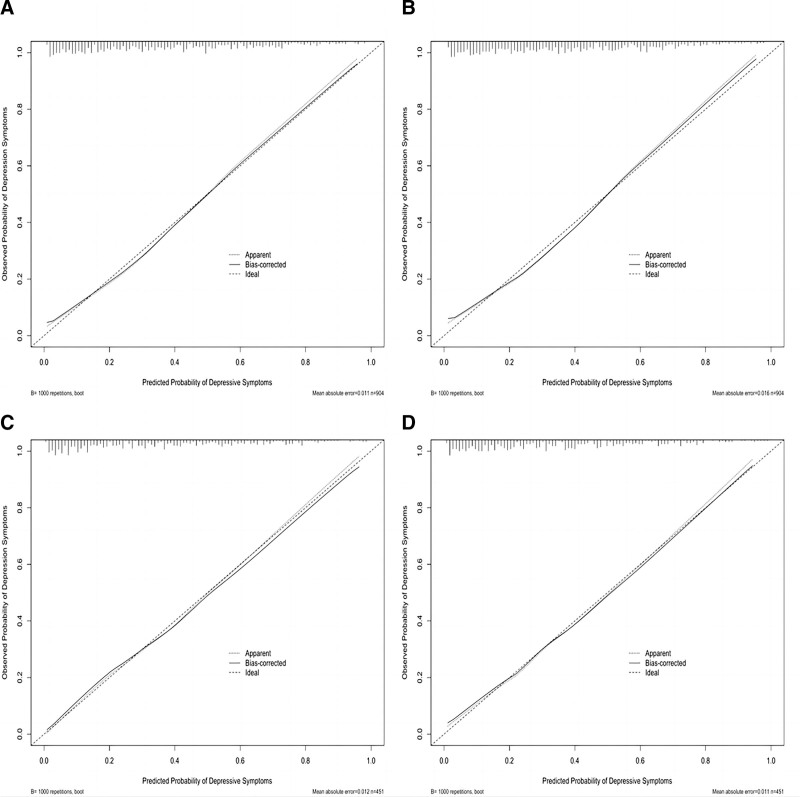
Calibration curves of the Nomogram prediction in the training set and validation set. (A) Calibration curves of Nomogram^1^ in the training set. The plot illustrates the agreement between the predicted probabilities of depression symptoms by Nomogram^1^ and the actual observed probabilities in the training set. (B) Calibration curves of Nomogram^2^ prediction in the training set. The plot demonstrates the alignment between the predicted probabilities of depression symptoms by Nomogram^2^ and the actual observed probabilities in the training set. (C) Calibration curves of Nomogram^1^ prediction in the validation set. The plot showcases the concordance between the predicted probabilities of depression symptoms by Nomogram^1^ and the actual observed probabilities in the validation set. (D) Calibration curves of Nomogram^2^ prediction in the validation set. The plot exhibits the correspondence between the predicted probabilities of depression symptoms by Nomogram^2^ and the actual observed probabilities in the validation set. Calibration curves are used to assess the performance and reliability of predictive models. They evaluate how well the predicted probabilities align with the actual probabilities of the outcome of interest. These calibration curves provide insights into the accuracy and calibration of the Nomogram predictions in both the training and validation sets, helping to assess the model’s performance in estimating the likelihood of depression symptoms.

**Figure 4.**
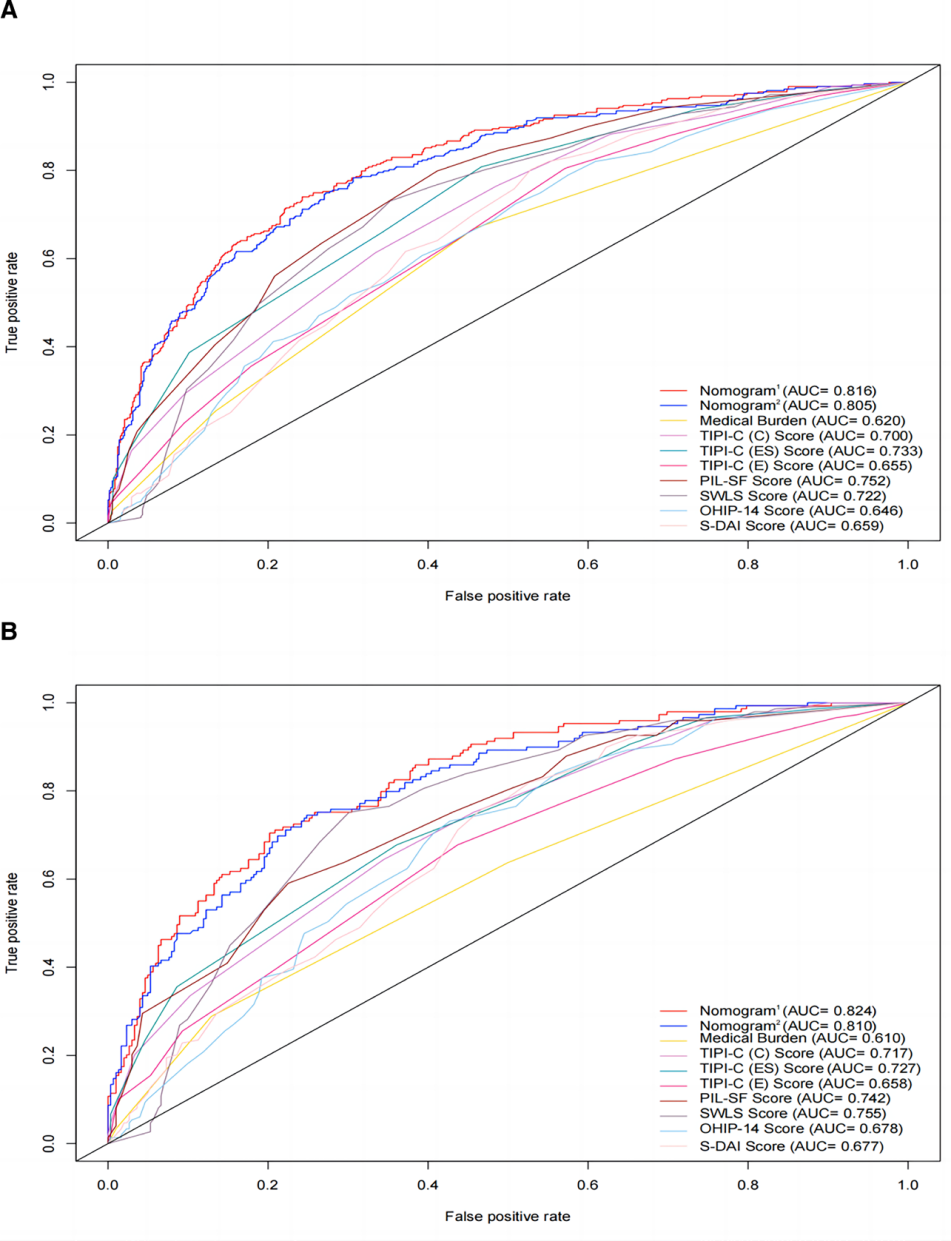
Receiver operating characteristic (ROC) curves for the prediction of depression symptoms in the training set and validation set. (A) ROC curves of the factors and Nomogram^1^ and Nomogram^2^ in the training set. (B) ROC curves of the factors and Nomogram^1^ and Nomogram^2^ in the validation set. DAI = The Stouthard’s Dental Anxiety Inventory, OHIP-14 = The Oral Health Impact Profile-14, PIL-SF = The Purpose in Life Test-Short Form, SWLS = The Satisfaction With Life Scale, TIPI-C (A) = Ten Item Personality Inventory (Agreeableness), TIPI-C (C) = Ten Item Personality Inventory (Conscientiousness), TIPI-C (E) = Ten Item Personality Inventory (Extroversion), TIPI-C (ES) = Ten Item Personality Inventory (Emotional Stability), TIPI-C (OE) = Ten Item Personality Inventory (Openness to Experiences).

Nomograms were developed to predict the risk of depressive symptoms using predictors such as medical burden, extraversion, conscientiousness, emotional stability, purpose in life, life satisfaction, oral health, and dental anxiety. The performance was evaluated by using the Area Under the Receiver Operating Characteristic Curve (AUC) and the Concordance index (C-index). The Area Under the Receiver Operating Characteristic Curve (AUC) of nomogram^1^ and nomogram^2^ (without oral health and dental anxiety) were 0.816 (95% CI: 0.788–0.845) and 0.805 (95% CI: 0.775–0.835) respectively (see Fig. 4). The Area Under the Receiver Operating Characteristic Curve (AUC) of each of the predictors are presented in Figure 4A. The Concordance index (C-index) of nomogram^1^ and nomogram^2^ for the training set were 0.816 and 0.805 respectively, indicating excellent predictive discrimination ability. In inclusion, the calibration curves show a high degree of consistency between predictions and actual observations (Fig. 3A and B).

### 3.4. Validation of nomogram^1^ and nomogram^2^ in the validation set

The training set’s data were used to construct the nomogram^1^ and nomogram^2^, while the validation set were used to retest the nomograms. The Area Under the Receiver Operating Characteristic Curves (AUC) and Concordance index (C-index) of both nomograms are presented in Figure 4A and B respectively. The calibration curve demonstrated a good agreement between both prediction models in the validation set (Fig. 3C and D).

### 3.5. Clinical utility of nomogram^1^ and nomogram^2^

**Figure 5.**
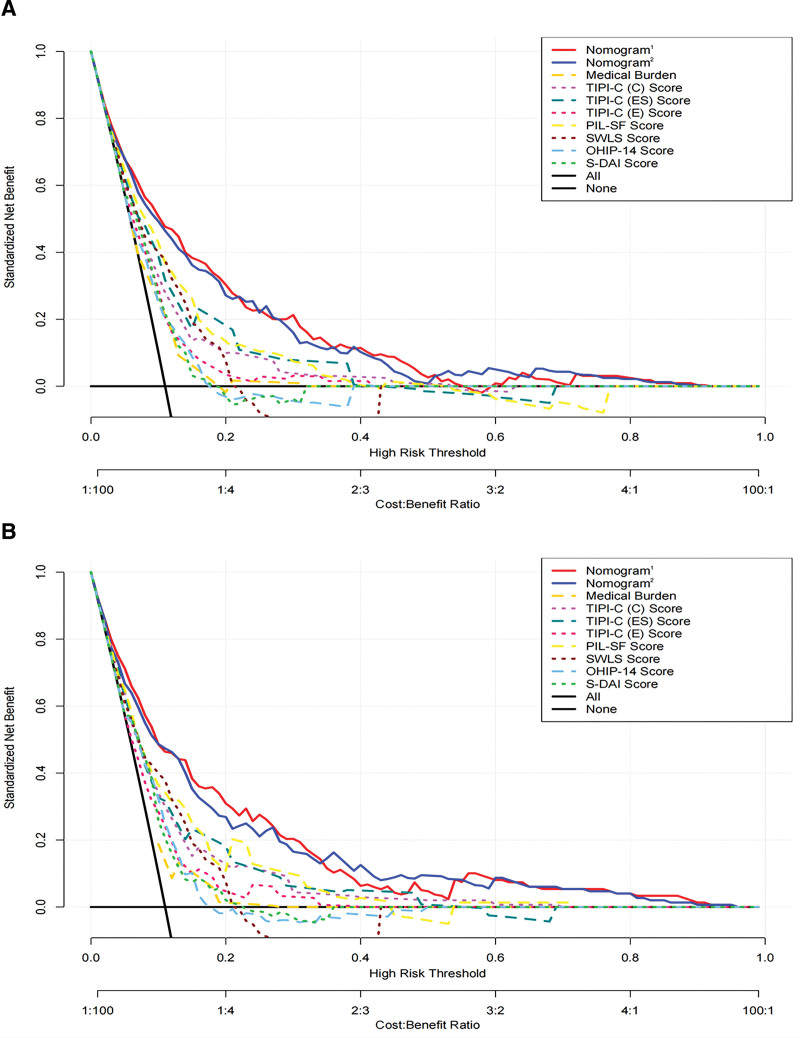
Decision curve analysis (DCA) of the Nomogram^1^ and Nomogram^2^ prediction in the training set and validation set. (A) DCA of Nomogram^1^ and Nomogram^2^ prediction in the training set. The decision curve analysis evaluates the clinical utility of Nomogram^1^ and Nomogram^2^ by assessing the net benefit across different threshold probabilities in the training set. It provides insights into the potential benefits of using the Nomograms in guiding clinical decision-making regarding the prediction of depression symptoms. (B) DCA of Nomogram^1^ and Nomogram^2^ prediction in the validation set. The decision curve analysis further examines the clinical utility of Nomogram^1^ and Nomogram^2^ by assessing the net benefit at various threshold probabilities in the validation set. This analysis helps to determine the practical value of the Nomograms in predicting depression symptoms in an independent dataset. Decision curve analysis (DCA) assesses the clinical impact and usefulness of predictive models by considering the balance between potential benefits and harms associated with different decision thresholds. The DCA curves in Figure 5 examine the net benefit of using Nomogram^1^ and Nomogram^2^ in predicting depression symptoms in both the training and validation sets. The analysis provides valuable information regarding the clinical applicability and potential advantages of incorporating the Nomograms into decision-making processes related to depression symptom prediction. DAI = The Stouthard’s Dental Anxiety Inventory, OHIP-14 = The Oral Health Impact Profile-14, PIL-SF = The Purpose in Life Test-Short Form, SWLS = The Satisfaction With Life Scale, TIPI-C (A) = Ten Item Personality Inventory (Agreeableness), TIPI-C (C) = Ten Item Personality Inventory (Conscientiousness), TIPI-C (E) = Ten Item Personality Inventory (Extroversion), TIPI-C (ES) = Ten Item Personality Inventory (Emotional Stability), TIPI-C (OE) = Ten Item Personality Inventory (Openness to Experiences).

**Figure 6.**
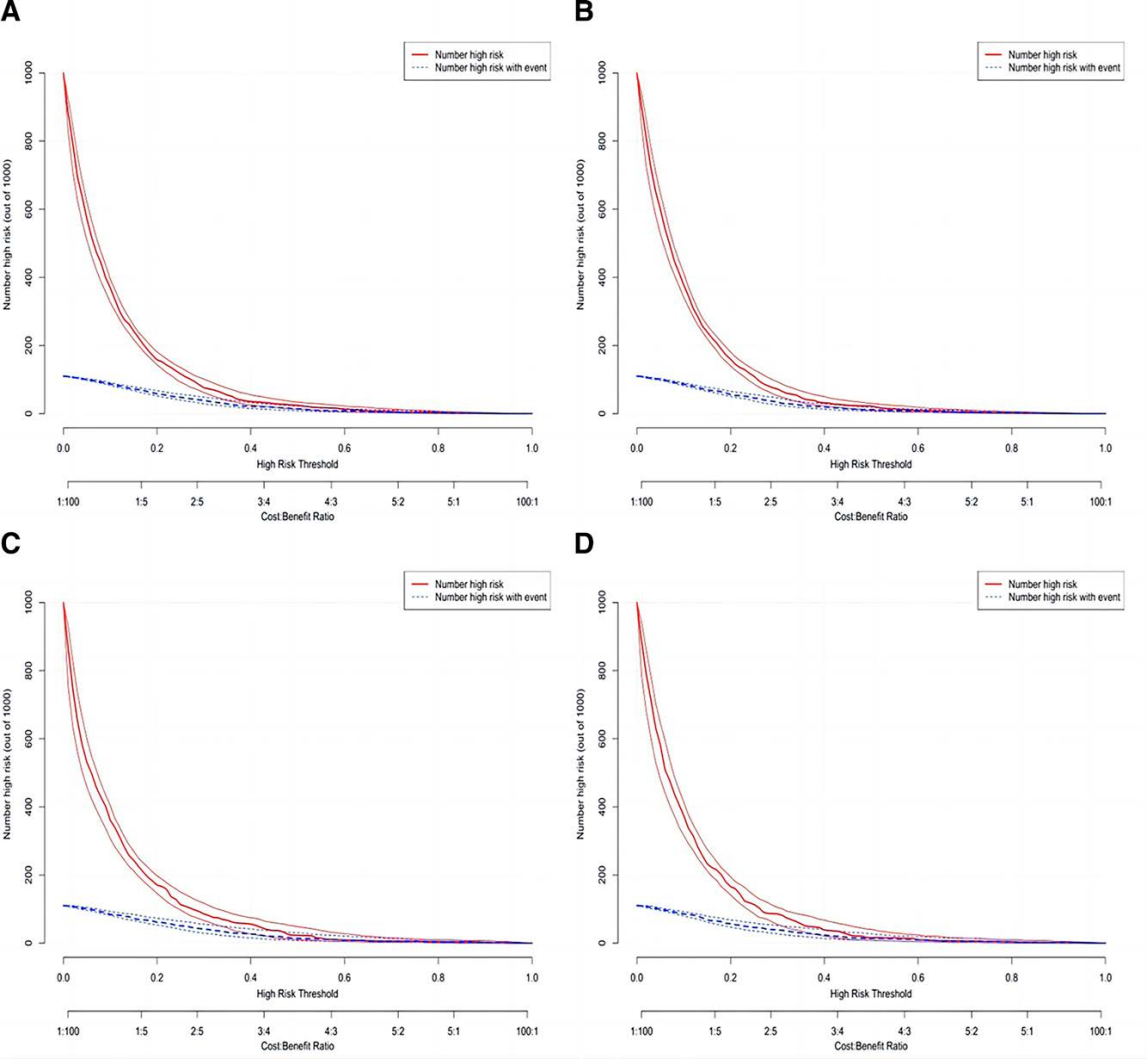
Clinical impact curves of the Nomogram^1^ and Nomogram^2^ prediction in the training set and validation set. (A) Clinical impact curves of Nomogram^1^ prediction in the training set. The clinical impact curves illustrate the net benefit of using Nomogram^1^ for guiding clinical decision-making regarding the prediction of depression symptoms in the training set. They provide insights into the potential clinical utility and impact of Nomogram^1^ across different threshold probabilities. (B) Clinical impact curves of Nomogram^2^ prediction in the training set. The clinical impact curves showcase the net benefit of using Nomogram^2^ for guiding clinical decision-making regarding the prediction of depression symptoms in the training set. They provide insights into the potential clinical utility and impact of Nomogram^2^ across different threshold probabilities. (C) Clinical impact curves of Nomogram^1^ prediction in the validation set. The clinical impact curves demonstrate the net benefit of using Nomogram^1^ for guiding clinical decision-making regarding the prediction of depression symptoms in the validation set. They provide insights into the potential clinical utility and impact of Nomogram^1^ across different threshold probabilities in an independent data set. (D) Clinical impact curves of Nomogram^2^ prediction in the validation set. The clinical impact curves illustrate the net benefit of using Nomogram^2^ for guiding clinical decision-making regarding the prediction of depression symptoms in the validation set. They provide insights into the potential clinical utility and impact of Nomogram^2^ across different threshold probabilities in an independent data set. The clinical impact curves assess the practical value and benefits of utilizing Nomogram^1^ and Nomogram^2^ in making informed decisions related to the prediction of depression symptoms. They provide an evaluation of the net benefit based on the predicted probabilities and can assist clinicians in choosing appropriate threshold probabilities for decision-making in clinical practice.

Decision Curve Analysis (DCA) was performed to assess the clinical utility of nomogram^1^ and nomogram^2^. Both nomograms demonstrated a higher net benefit in identifying depressive symptoms compared to any single predictor in both the training set and validation sets (Fig. 5A and B). Furthermore, the clinical impact curves (CIC) based on Decision Curve Analysis (DCA) indicated that the predicted probabilities from nomogram^1^ and nomogram^2^ were well-aligned with the actual probabilities in the training set (Fig. 6A and B), as well as in the validation set (Fig. 6C and D).

### 3.6. Comparison between nomogram^1^ and nomogram^2^

In total group, the Net Reclassification Improvement (NRI) of Nomogram^2^ compared to Nomogram^1^ in predicting the risk of depressive symptoms was −0.035 (95% CI: −0.008 to 0.078, *P* = .108). The continuous Net Reclassification Improvement (NRI) of −0.374 (95% CI: −0.264 to −0.484, *P* < .001), and the Integrated Discrimination Improvement (IDI) was −0.021 (95% CI: −0.013 to −0.029, *P* < .001) for forecasting the risk of depressive symptoms. These outcomes suggested that Nomogram^1^ demonstrated superior forecasting ability than Nomogram^2^.

## 4. Discussion

Oral disease is known to affect not only the physical and mental health of patients,^[43]^ but poor oral health can also have a negative impact on their lives.^[44]^ If they have to deal with dental anxiety at this time, the risk of depression will increase.^[45]^ Previous study also found that poor dental health habits may mask an individual’s mental and physical activity, and it may also be a cause of depressive symptoms.^[46]^ In this study, about 34.83% of patients who came to the department of stomatology had mild or more depressive symptoms, which is consistent with previous studies.

For dentists, it is of great significance to use reliable tools to evaluate the mental health of patients in dental treatment.^[47]^ Therefore, we incorporated the relevant risk factors into an easy-to-use nomination chart to facilitate dental clinicians and patients facing the challenge of managing depressive symptoms. Of course, with the rise of clinical prediction model, they are more and more widely used in various fields, in the prediction of depression applications also gradually increased. However, the majority of research attention remains focused on the predictive role of physiological indicators on depressive symptoms, and these factors were also screened for influences that could be included in the Nomogram by a combination of multivariate logistic regression and univariate logistic regression.^[48–50]^ We wanted to build on this base as, on the one hand, we wanted to screen variables by combining machine learning methods (e.g., LASSO regression) and multivariate logistic regression to better identify independent predictors. On the other hand, we combined demographic variables from previous studies that were much this included in the Nomogram and on this basis selected some psychological variables and behavioral factors that may have an impact on depressive symptoms to be included in the model. To our knowledge, few studies have included the use of psychological factors (purpose in life, subjective well-being, and life satisfaction) in predictive models of depression risk. However, in previous studies constructing predictive models, we found that these variables reflect the values, attitudes and behavioral patterns of the individual. Combined with the results of previous studies, we believe that incorporating these factors into the Nomogram can more accurately determine the potential risk of patients developing depressive symptoms. In addition, this study included patients who came to the dentistry department as the study population, and oral-related factors (dental anxiety and oral health) should be taken into consideration in order to better develop a predictive model of depressive symptoms adapted to dentistry patients.

Based on the findings of Nomogram^2^, we found that medical burden, personality traits (Extraversion, Conscientiousness and Emotional Stability), purpose in life, and life satisfaction were all independent predictors of depressive symptoms. First, medical burden is a significant risk factor, and the higher the medical burden felt by patients during their dental visits, the higher the potential risk of developing depressive symptoms. This may be because patients who feel a high burden of medical care during their visits are more likely to face financial difficulties. On the one hand, economic difficulties are an important source of stress for patients and they increase the risk of depressive symptoms.^[51]^ On the other hand, low income and poor economic status not only lead to more stress and hardship for these patients, but also act as a barrier for these patients to seek mental health service.^[52]^ Second, certain personality traits have long been known to increase a patient’s risk of developing depressive symptoms.^[53]^ Specifically, extraversion negatively predicted the probability of depressive symptoms.^[54]^ Extraversion (it is a trait characterized by sociability, high energy levels, and appreciation of social interactions) may have a range of positive effects on an individual’s mental health.^[55,56]^ Individuals who are extroverted are accustomed to relying on the social support system between them to solve problems and are more active in seeking help when they experience problems, which can mitigate the negative effects of various stressors on them.^[57]^ Meanwhile, Consciousness is a face-level trait, which initially has 6 fundamentals: diligence, order, autonomy, accountability, heritage and ethics. From this angle, people with strong consciousness are characterized by good organization, diligence, perseverance, prudence, reliability and foresight, while people with poor consciousness are chaotic, reckless, impulsive, unreliable and inconsistent.^[58]^ Poorer organization, planning, discipline and quite rushed actions (typical of people with low self-awareness) may lead to pressure in a variety of areas, such as under performance at job, in school or in interpersonal connections.^[59]^ Hence, individuals with low self-awareness may be prone to failure, which may lead to psychopathology that can maintain or increase depressive symptoms.^[60]^ And emotional stability had the most pronounced effect on depressive symptoms in patients with oral diseases of all personality traits included in the Nomogram. Emotional stability is a personality trait that includes the ability to regulate emotions, control impulses, and deal with life challenges.^[61]^ Empirical studies have shown that emotional stability is positively correlated with positive emotions,^[62]^ and negatively correlated with negative emotions, anxiety and depression.^[10]^ Emotional stability has a protective effect on patients’ emotional well-being - perhaps by coping with or regulating their emotions. Although dentistry patients may face stress from a variety of sources, emotionally stable patients may have the ability to adjust their emotional responses in stressful situations; therefore, they demonstrate better emotional functioning (fewer depressive symptoms). Second, the relationship between life satisfaction and depressive symptoms has been demonstrated, and life satisfaction is also considered to be an accurate predictor of depressive symptoms.^[63]^ Life satisfaction comes primarily from an individual’s assessment of past experiences, which can also provide clues and resources for predicting a positive or negative future.^[64]^ On the one hand, individuals with high life satisfaction tend to accept the status quo and face the challenges in life positively. High life satisfaction can expand and build the resources needed for the future. Individuals with high life satisfaction will encounter relatively ideal situations, in which their resources are sufficient and their goals are easy to achieve.^[65,66]^ To put it differently, greater life satisfaction means that life is going well, individuals have more active moods and are kept in a state free from imminent risk and recent losses, through which they can break and unwind to reestablish their energy and broaden their resources, pursue new future objectives and be well prepared for coming opportunities and challenges.^[67]^ On the other hand, individuals with low life satisfaction usually have a more negative attitude towards life and are more dissatisfied with the status quo, which is more likely to affect their mood in life and increase the risk of depressive symptoms. For example, retrospective emotions can affect an individual’s expectations of future events,^[68]^ and past failures can lead to a decrease in the individual ‘s positive expectations of the future.^[69]^ Finally, the results demonstrated that the numerical range of purpose in life is wide and has good applicability for predicting high risk of depressive symptoms. To be franker, purpose in life had a significant effect on depressive symptoms in patients with oral diseases, which is in accordance with the results of previous surveys.^[70]^ The purpose in life can be framed as a fundamental objective that is used to help self-organize and inspire a concrete target, provoke action, and provides rewarding evaluation.^[71]^ Having a purpose in life and valuable goals, giving direction to one’s actions and providing a sense of direction are mentioned as a key element of a meaningful life.^[72]^ Individuals with purposes in life have clear expectations for the future, which allows them to adjust to difficulties and setbacks as soon as possible and avoid being troubled by negative emotions for a long time. In contrast, individuals who lack a purpose in life are more likely to lose motivation and information due to setbacks in their lives, which causes their mood to be negatively affected and the probability of depressive symptoms to increase.^[73]^

To better identify patients with dental diseases at high risk of predicting depressive symptoms, we included oral-related factors (dental anxiety and oral health) in Nomogram^1^. For Nomogram^1^, the poorer the oral health status, the higher the level of dental anxiety and the increased risk of depressive symptoms. On the one hand, although depression and dental anxiety often occur together, the hallmark symptoms of depressive symptoms are emotional (hopelessness and sadness), for example, poor appetite, fatigue and sleep, whereas dental anxiety is often an excessive fear during dental visits, referring to the perceived threat of this possible event,^[74]^ although these 2 psychological disorders exhibit different symptoms, it is certain that the persistence of dental anxiety leads to a constant generalization of this negative emotion,^[75]^ which gradually transforms into a negative impact on the individual’s life, and depressive symptoms emerge. On the other hand, the degree of oral health impact reflects how much patients are disturbed by oral problems, which are multifaceted and can affect not only their psychological state but also their daily life, and therefore the degree of correlation with depressive symptoms is self-explanatory. Given the relevance to the outcome, we used only these 2 relatively objective oral-related indicators to predict the risk of depressive symptoms. Comparing with Nomogram^2^, Nomogram^1^ was not only superior in predicting high-risk groups, but could also be reliably applied to the patients with oral disease. Thus, we conclude that in addition to the included indicators in Nomogram^2^, we should also focus on dental anxiety and oral health of dentistry patients when considering the factors influencing depressive symptoms. The inclusion of these 2 indicators would be of great value in identifying depressive symptoms in dentistry patients, and the Nomogram developed based on the above indicators could also provide a more accurate measurement tool for dentists to identify depressive symptoms in patients with dental disease.

There are several limitations to be acknowledged in this study. Firstly, the study population consisted of patients attending the dentistry department, without being divided according to specific diseases or symptoms, leading to a high degree of heterogeneity. However, it is important to note that dental care is costly in China, and most patients who seek dental treatment have varying degrees of oral problems. Therefore, we believe that the patients attending the dentistry department can be considered representative of the population with oral health issues. Secondly, the sample size of the study warrants consideration. Although efforts were made to include patients of all ages, it remains uncertain whether they fully represent the entire population visiting the dentistry department. A larger and more diverse sample could provide a more comprehensive understanding of the relationship between oral diseases, depressive symptoms, and associated risk factors. Furthermore, the assessment of depressive symptoms in this study was based on a single scale, namely the PHQ-9 scale. While the PHQ-9 is a widely used and validated tool for assessing depressive symptoms, it is important to acknowledge that depression is a complex and multifaceted condition that may require a comprehensive evaluation using multiple scales. Considering a patient to have depressive symptoms only when all scales indicate the presence of symptoms might provide a more comprehensive and accurate assessment of depression. In future research, it would be beneficial to consider a more diverse range of patients with specific oral diseases or symptoms, increase the sample size, and employ multiple assessment scales to obtain a more comprehensive understanding of the relationship between oral diseases, depressive symptoms, and associated risk factors in dentistry patients.

## Author contributions

**Conceptualization:** Jimin Zhang, Heli Lu.

**Data curation:** Jimin Zhang, Zewen Huang, Heli Lu.

**Formal analysis:** Jimin Zhang, Zewen Huang, Lejun Zhang, Heli Lu.

**Investigation:** Zewen Huang, Lejun Zhang, Heli Lu.

**Methodology:** Jimin Zhang, Zewen Huang, Wei Wang, Lejun Zhang.

**Project administration:** Jimin Zhang, Lejun Zhang.

**Resources:** Jimin Zhang, Wei Wang, Heli Lu.

**Software:** Jimin Zhang, Wei Wang, Heli Lu.

**Supervision:** Jimin Zhang.

**Validation:** Jimin Zhang.
